# Symptomatic Pericardial Cyst: An Atypical Case of Pleuritic Chest Pain

**DOI:** 10.5811/cpcem.2019.5.42601

**Published:** 2019-07-01

**Authors:** T. Douglas Sallade, Chadd K. Kraus, Lisa Hoffman

**Affiliations:** Geisinger Medical Center, Department of Emergency Medicine, Danville, Pennsylvania

## Abstract

Pericardial cysts were first described in 1837 as diverticula extending from the pericardium. They are rare and frequently asymptomatic. Symptomatic presentations may be similar to more common causes of chest pain or dyspnea such as acute coronary syndrome or pulmonary embolism. Emergency physicians should consider mediastinal mass, and in this case pericardial cyst, in the differential diagnosis of chest pain because of the risk for tamponade, sudden cardiac death, or other life-threatening complications. Here, we describe a novel presentation of a pericardial cyst presenting as atypical chest pain.

## INTRODUCTION

Pericardial cysts were first described in 1837 as diverticula extending from the pericardium.^1^ They are usually asymptomatic and are incidentally found on chest radiograph (CXR) as a well-circumscribed opacity over the right heart border.^2^ One large study using CXRs suggested a prevalence of pericardial cysts as one in 100,000 people.^3^ We describe a case of a pericardial cyst causing pleuritic chest pain in a patient presenting to an emergency department (ED).

## CASE REPORT

A 43-year-old female presented to the ED of an academic medical center for sudden onset chest pain. The pain was severe and pleuritic, radiating over her anterior chest and through to her shoulder blades. The pain worsened when lying flat, making her dyspneic. Associated symptoms included irregular, pounding heartbeats and occasionally a “whooshing” sensation in her chest. Review of systems was otherwise negative.

For approximately six months prior to ED presentation, she had presented to several urgent care centers for respiratory symptoms including “not getting air” and cough. Her symptoms were attributed to bronchitis or asthma and treated with azithromycin and prednisone with variable relief. Her past medical history included asthma, gastroesophageal reflux, and migraine headaches.

On arrival to the ED, vital signs included a blood pressure of 142/77 millimeters of mercury, heart rate of 89 beats per minute, respiratory rate of 16 breaths per minute, oxygen saturation of 98% on room air, and temperature of 36.7 degrees Celsius. Physical exam revealed an anxious-appearing female of stated age. Cardiac exam revealed regular rhythm, with first and second heart sounds present, and no murmurs, rubs, or gallops. Pulmonary exam revealed clear lungs, normal effort, and was significant for pain on deep inspiration. The remainder of the physical exam was unremarkable.

**Image 1:**
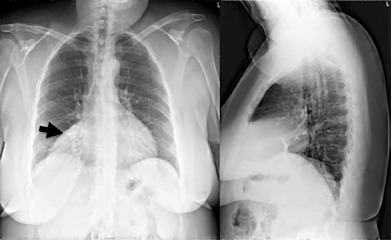
Chest radiograph: Anterior-posterior and lateral films demonstrating anterior mediastinal opacity over the right heart border (arrow).

The complete blood count, basic metabolic panel, and high-sensitivity troponin were unremarkable. D-dimer was elevated (0.86 micrograms per milliliters (ug/ml) fibrinogen equivalent units, normal <0.50 ug/ml fibrinogen equivalent units). Electrocardiogram showed normal sinus rhythm with a non-specific T-wave inversion in lead III. CXR was significant for a large opacity over the right mediastinal border (Image 1). C-reactive protein (15 milligrams per liter (mg/L), normal 0–5 mg/L) and erythrocyte sedimentation rate (31 millimeters per hour (mm/h), normal 0–20 mm/h) were ordered following evidence of opacity on CXR.

**Image 2:**
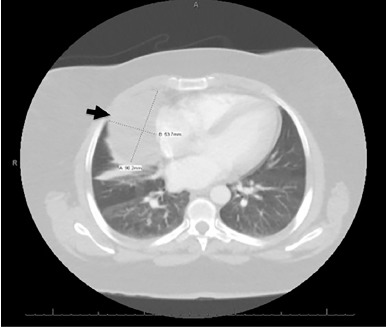
Axial slice of computed tomography (CT) of the chest (pulmonary embolism protocol): CT showing pericardial cyst (arrow).

The elevated D-dimer prompted computed tomography (CT) to evaluate for pulmonary embolism and to better define the opacity seen on chest radiograph. The CT was negative for pulmonary embolism but revealed a large pericardial cyst (Image 2).

**CPC-EM Capsule**

What do we already know about this clinical entity?

Pericardial cysts are rare mediastinal masses that are usually asymptomatic but can be complicated by arrhythmia, tamponade, and sudden cardiac death.

What makes this presentation of disease reportable?

Among symptomatic pericardial cysts, pleuritic pain is not a common presenting symptom. This cyst was found to be infected.

What is the major learning point?

Symptomatic pericardial cysts are uncommon presentations of a rare condition. Urgent echocardiography and surgical excision may be necessary in their management.

How might this improve emergency medicine practice?

It is important to consider mediastinal mass and, more specifically, pericardial cyst in the differential diagnosis of chest pain.

Transthoracic echocardiogram was negative for tamponade physiology. The patient was hospitalized and two days later taken for thoracoscopy and cyst excision. Pathology interpreted the tissue sample as a mesothelial-lined cyst with marked acute and chronic inflammation consistent with infected pericardial cyst containing purulent exudate. Fluid gram stain and cultures were negative. Blood cultures were not obtained and the patient did not receive antibiotics. Cytology was benign and noted intense acute inflammation of unknown etiology. The patient required no further treatment, had no further symptoms, and had no complications at her postoperative follow-up one month later.

## DISCUSSION

This is a novel presentation of a rare condition. Most pericardial cysts are asymptomatic. Ranging in size from 1–15 centimeters, 70% appear as a round opacity at the right cardiophrenic angle, while approximately 20% are found on the left heart border, and the remaining 10% are found either in the superior or posterior chest.^2^,^4^ Chest pain, dyspnea, or cough are common presentations of symptomatic cysts. More uncommon presentations are documented and include complications such as atrial fibrillation, sudden cardiac death, right heart failure, cyst rupture, airway obstruction, or cardiac tamponade.^5^ After identifying a cyst, further diagnostic evaluation includes serial transthoracic echocardiograms for asymptomatic patients. Symptomatic patients may require aspiration or surgical resection of the cyst. 5

This patient’s presentation of pericardial cyst was atypical for several reasons. First, she presented with pleuritic chest pain, an uncommon symptom of pericardial cysts. Second, the pathology analysis of the cyst and purulent fluid was consistent with infection. Pericardial cysts are classically described as “spring water” cysts because they usually contain clear fluid.^1^ Lastly, the large size of this cyst may have contributed to the pleuritic nature of the patient’s chest pain. It is reasonable to consider that further enlargement or rupture of the cyst could have put her at risk for complications such as tamponade or intrathoracic infection. Excision of this large, symptomatic, and inflamed cyst resolved the patient’s symptoms in-hospital and at her one-month follow-up appointment.

## CONCLUSION

Symptomatic pericardial cysts are uncommon. They may masquerade as more common causes of chest pain, pleuritic chest pain, or dyspnea such as acute coronary syndrome or pulmonary embolism. Emergency physicians should include pericardial cysts in the differential diagnosis of pleuritic and other chest pain presentations. Careful exclusion of more common etiologies is necessary; however, consideration of mediastinal mass and more specifically pericardial cyst in the differential diagnosis is important because of the risk for tamponade, sudden cardiac death, or other life-threatening complications. Our patient had a definitive diagnosis and surgical excision with no complications from the pericardial cyst.
